# Systemic mechanism of Panax noteginseng saponins in antiaging based on network pharmacology combined with experimental validation

**DOI:** 10.1002/ibra.12165

**Published:** 2024-06-01

**Authors:** Yang‐Yang Zhao, Li‐Xia Yang, Shuang‐Yu Que, Lei‐Xing An, Abeer A. Teeti, Shun‐Wu Xiao

**Affiliations:** ^1^ Department of Neurosurgery Affiliated Hospital of Zunyi Medical University Zunyi China; ^2^ Department of Chemistry, School of Science Hebron University Hebron Palestine

**Keywords:** aging, bioinformatics analysis, molecular docking, network pharmacology, Panax notoginseng saponins

## Abstract

This study aims to investigate the systemic mechanism of Panax notoginseng saponins (PNS) in antiaging using network pharmacology combined with experimental validation. String database and Cytoscape3.7.2 were used to perform the protein–protein interaction (PPI) and construct genes network. The key target genes were analyzed using gene ontology (GO) and Kyoto Encyclopedia of Genes and Genomes (KEGG). Then, the aging‐related genes were verified by reverse‐transcription polymerase chain reaction in SAM‐P/8 mice, and performed molecular docking with the main components of PNS. Moreover, it produced cluster between Hub genes and differential genes. A total of 169 crossover genes were obtained, and the results of GO and KEGG indicated that the antiaging effect of PNS was mediated by apoptosis, cancer, and neurodegeneration and that five of the eight Hub genes had good binding activity with the main components of PNS. In addition, animal experiments reported that MAP2, MAPKK4, RAB6A, and Sortilin‐1 have different levels of expression in the brain tissues of aging mice, and bind well docking with the main active components of PNS. However, there was no crossover between the 169 PNS intersecting genes and the four differential genes, while they yielded a link from PPI in which MAP2K4 was only linked to AKT1 and CASP3; MAP2 was only linked to AKT1 and CASP3; RAB6A was only linked to AKT1; but Sortlin‐1 did not link to the Hub genes. In summary, the antiaging effect of PNS is associated with the eight Hub genes and four differential genes. All of them consist of a cluster or group that is possibly related to the antiaging effect of PNS.

## Graphical abstract

In this study, we utilized network pharmacology and molecular docking techniques to explore the main targets and possible mechanisms of Panax notoginseng saponins (PNS) antiaging. By querying PNS‐related target genes and aging‐related genes, the first eight core genes were constructed and gene ontology, Kyoto Encyclopedia of Genes and Genomes analysis, and molecular docking were performed. These results indicate that the antiaging effect of PNS is achieved through the synergistic effect of apoptosis, cancer, neurodegeneration, and other pathways. However, unlike previous studies, animal experiments in the PNS group revealed a decrease in the expression of MAP2, MAPKK4, Rab6A, and Sortilin‐1. They may be new antiaging targets.

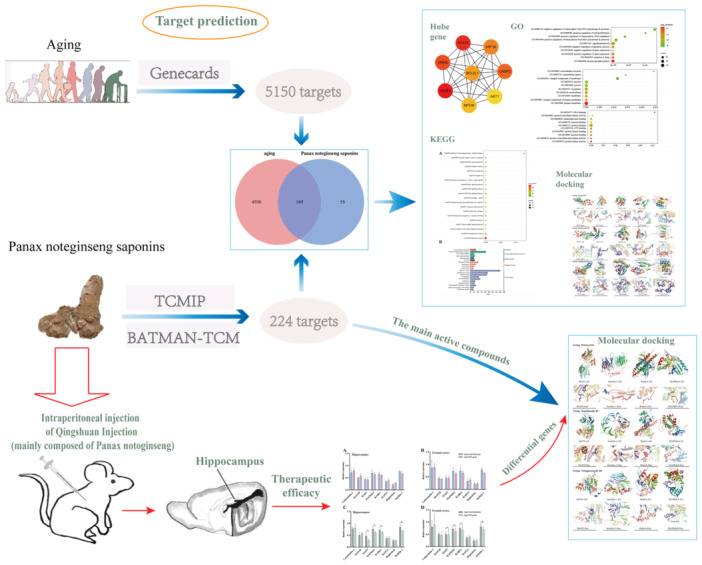

## 1 INTRODUCTION

Aging is an unavoidable spontaneous process of organisms over time. As a complex natural phenomenon, it is compared with structural degradation and functional decline, decreased adaptability and resistance. In the process of aging, there will be some damages caused by the decline of body function, such as the increased incidence of some chronic diseases and neurodegenerative diseases,^1^ which has brought a lot of pain to the life of the elderly. The decline of cognitive ability is a common manifestation of aging,^2^ which is mainly due to the damage of the hippocampus.^3^ Brain aging is most obvious during aging, especially encountering the cognitive decline caused by hippocampal aging.^4^ The neurological function of the adult hippocampus decreases with age, and premature hippocampal nerve damage occurs in the hippocampus of patients with Alzheimer's disease (AD).^5^ The aging body is more likely to cause more serious damage. A study has found that after the body is infected, the aging body will amplify the neuroinflammation caused by the infection,^6^ which brings more serious damage to the body with weakened immunity. According to the results of statistical analysis, the aging level of the world's population is rising, and it is expected that by 2050,^7^ the proportion of the world's population aged 60 or above will reach about 22%. With the development of aging population, the incidence of chronic diseases such as coronary heart disease, hypertension, AD, Parkinson's disease (PD) will increase, and the occurrence of these diseases will bring a huge burden to society. As one of the countries with an aging population, China exhibited high prevalence of AD, which also brings a great burden to our social life.^8^ Hence, the quest for effective antiaging strategies is paramount. Notably, Chinese Medicine offers promising potential in the realm of antiaging since many Chinese herbs contain potent antioxidants and anti‐inflammatory compounds that can help combat the oxidative stress and inflammation associated with aging. Therefore, the exploration of Chinese medicine for the prevention and management of age‐related conditions warrants further attention and investigation.

Notoginseng, as a Chinese herbal medicine, has a long history in China and is widely used for various diseases. Panax notoginseng saponins (PNS) is an active ingredient extracted from Notoginseng and is mainly composed of ginsenoside Rb1, ginsenoside Rg1, and notoginsenoside R1.^9^ At present, the pharmaceutical preparations of PNS mainly include Thrombus Scavenger Injection and Blood Embolization, which are mainly used for the treatment of collateral congestion and obstruction, stroke hemiplegia, chest pain, central retinal vein occlusion, etc. Many studies showed that PNS has good antioxidant effects,^10^, ^11^ antidepressant effect,^12^ and also has the effect of improving neurodegenerative diseases and neuroprotection.^13^ It is certain that PNS plays a certain role in regulating the cognitive dysfunction caused by hippocampal dysfunction.^14^ According to the role of PNS and the current research, we believe that PNS may have the effect of improving some lesions caused by the decline of body function during aging. However, the network mechanism of PNS in antiaging is still unclear.

Network pharmacology has the advantage to analyze the network of biological systems and find specific key signal targets for multi‐target drug molecular design.^15^ The emergence of network pharmacology has provided great help for the application of Chinese herbal medicine and the development of new drugs. On the premise of ensuring the research effect, it not only improves the research efficiency but also reduces the research cost. Here, we will use the network pharmacology method to study the key points and pathways of PNS in the antiaging process and validate novel gene lists based on animal experiments.

## 2 METHODS

### 2.1 Screening of PNS‐related genes and aging‐related genes

The Chinese medicine system pharmacology database and analysis platform (TCMIP) database (http://www.TCMIP.cn/TCMIP/index.php/home/) and the Chinese medicine molecular mechanism bioinformatics analysis tool (BATMAN‐TCM) database (http://BioNet.ncpsb.org/BATMAN-TCM/) were used to screen the active components of PNS with the key word “Notoginseng”. Finally, through the results intersection of the two databases, the active compounds in PNS were obtained, which were, respectively, Notoginsenoside R4, Ginsenoside Rb1, Notoginsenoside R3, Notoginsenoside R1, Notoginsenoside A, Acetophenone, Notoginsenoside R2, Sanchinoside B1, Cyclododecanone, Stigmasterol, Panaxytriol, and Cuparene. Then, the simplified molecular input line entry system (SMILES) information corresponding to each component was imported into the Swiss Target Prediction database (http://www.swisstarget prediction. ch/), and the targets corresponding to all the active components with the binding probability greater than 0 were screened to obtain the genes related to PNS. Genes related to aging were obtained using the GeneCards (https://www.genecards.org/) database with “aging” as a search term.

### 2.2 Drug‐disease core genes analysis and protein–protein interaction network construction

The screened PNS and aging‐related targets were uploaded to the website of Lianchuan Biological Cloud Platform (https://www.omicstudio.cn/tool/) for Venny map, and intersection targets of PNS and aging were found. To obtain more accurate data, we sequenced these intersection target genes according to Cytoscape3.7.2 and selected the first 8 as the required core targets, then the website String (https://cn.string-db.org/) was used to perform protein–protein interaction (PPl) analysis by importing the intersection gene and selecting “Homo sapiens” in the “Organisms” option, with the parameter of “minimum required interaction score” as 0.400. After analysis, PPI network was generated according to the degree values.

### 2.3 Gene ontology and Kyoto Encyclopedia of Genes and Genomes

In this study, the intersecting target of PNS and aging was analyzed by Kyoto Encyclopedia of Genes and Genomes (KEGG) and Gene Ontology (GO) analysis. Annotation of genes and gene products was conducted according to biological process (BP), cellular component (CC), and molecular function (MF). KEGG was a useful resource for systematic analysis of information on gene function and related advanced genomic functions. Therefore, to further clarify the function of the selected crossover genes and their role in the signal transduction pathway, GO functional analysis and KEGG pathway enrichment analysis were performed using DAVID's database (https://david.ncifcrf.gov), and the P‐value and fold enrichment values were obtained.

### 2.4 Animals

The animal protocol for this study has been approved by the Animal Protection and Welfare Committee of Kunming Medical University (No. kmmu20230340). Healthy and clean SAM‐P/8 mice (25 ± 2 g) were purchased from the Experimental Animal Center of Tianjin University of Chinese Medicine. Mice were housed in control rooms at 22 ± 2.0°C and 50 ± 5% humidity under approximately 12 h of light per day with free access to food and water. Twenty SAM‐P/8 mice were randomly divided into four groups (*n* = 5/group): adult control group, adult PNS group, aged control group, and aged PNS group.

### 2.5 Drug injection

The Thrombus Scavenger Injection (purchased from Yunnan Plant Pharmaceutical Co., Ltd.), composed of PNS, was diluted with 0.9% normal saline for medical use at the ratio of 5 mg/mL and injected intraperitoneally at the dose of 60 mg/kg, at 12:00 noon each day, one time per day, for successive intraperitoneal injection for 14 days. The adult control group and the aged control group were intraperitoneally injected with the same dose of normal saline, at 12:00 noon, once a day for 14 consecutive days.

### 2.6 Sampling

After all the animals were raised normally for 14 days, the mice were deeply narcotized with 5% isoflurane, and then they were fixed on the foam board with the thoracic cavity and abdominal cavity exposed. The remaining needle with the needle core removed was inserted into the ventricle along the lower edge of the left ventricle until it was fixed behind the aortic arch, and blood reflux could be seen at the needle tip. After the right atrial appendage was cut open and a large amount of blood outflow was observed, about 200 mL pre‐cooled sterile normal saline (4°C) was injected into the liver for whitening until the outflow from the right atrial appendage was clear. After the frontal cortex and hippocampus tissues were taken out by craniotomy, they were quickly placed in an environment of −80°C.

### 2.7 Reverse‐transcription polymerase chain reaction

It was reported that eight genes were closely associated with aging.^16^ Therefore, reverse‐transcription polymerase chain reaction (RT‐PCR) was utilized to validate their expression changes in different groups, in which, genes with significant expression changes were regarded as differential genes. Total RNA of frontal cortex and hippocampus was prepared by tissue homogenization, then isolation of RNA, precipitation of RNA, washing of RNA, and dissolution of RNA were performed, ensuring high sample purity before proceeding to the next experimental step. Next, 1.5 µg RNA samples were taken and cDNA was synthesized according to the instructions of RevertAidTM First‐strand cDNA Synthesis Kit. After the first strand of the synthesized cDNA can be directly used for the PCR amplification reaction, the specific steps are as follows. The RNA sample volume required was calculated and added to a 0.5 mL PCR tube kept on ice, along with 1 µL Oligo (dT) primer and DEPC‐treated water to a total volume of 12 µL. The mixture was gently shaken and centrifuged for 3–5 s to ensure uniform mixing. The samples were incubated at 70°C for 5 min, cooled in an ice bath, and centrifuged slightly. In the ice bath, they were mixed with the following reactants: 4 µL of 5× Reaction Buffer, 1 µL of RibolockTM Ribo Nuclease Inhibitor (20 g/L), and 2 µL of 10 mM dNTP Mix. After being shaken gently and centrifuged slightly for 3–5 s to mix the reactants, they were incubated at 37°C for 5 min. Subsequently, 1 µL of RevertAidTM M‐Mulv ReversE TRANSIPTASE was added to a final reaction volume of 20 µL. The samples were incubated at 42°C for 60 min, and put on by heating at 70°C for 10 min to terminate the reaction, followed by ice bath cooling. β‐actin and eight aging‐related genes were amplified according to the instructions of 2× PCR Master Mix Kit. The total volume of each reaction was 20 µL: 10 µL of 2× PCR Master Mix, 8 µL PCR Water nuclease‐free, 0.5 µL upstream primer, 0.5 µL downstream primer, and 1 µL reverse transcription cDNA template. Thermal cycle parameters: denaturation at 94 C for 5 min, denaturation at 94°C for 1 min, annealing for 1 min (Table 1) for annealing temperature corresponding to specific genes, extension at 72°C for 1 min, and extension at 72°C for 10 min after 35 cycles. Finally, 2 μL amplification products were taken, and the PCR products were detected by 1% agarose gel electrophoresis. For analysis, the gel images were taken under the ultraviolet mode of BIO‐GEL Imaging System, and the gray values of each related gene band were measured. The gray scale of electrophoresis bands was analyzed with Quantity One software, and the ratio of gray scale values of each target gene band to β‐actin was calculated.

**Table 1:** Upstream and downstream prim sequences of each gene, anneal temperature, RT‐PCR product length.

Genes	Primer sequence	Renaturation temperature (°C)	Product length (base pair)
MAP2	Sense 5′‐AGGAAGCAGCAAGTGGTGAC‐3′	53	427
	Antisense 5′‐TTTGGAGGAGTGCGGATG‐3′		
Sortilin‐1	Sense 5′‐CGCTACCGCAAAGAACAA‐3′	52	376
	Antisense 5′‐GGAAGCAAGCCCAGTGAA‐3′		
RAB6A	Sense 5′‐GTCCTTGATCACCCGATTC‐3′	52	465
	Antisense 5′‐TCCTGTGTGCTTTCCATTC‐3′		
MAPKK4	Sense 5′‐ATTGCCCATACATTGTTCAGT‐3′	51	398
	Antisense 5′‐ATCAGAGCGGACATCATACC‐3′		
MAP1B	Sense 5′‐TGCCCGCCATAAACTGC‐3′ Antisense 5′‐GGTGGGTGGTGCTTAGGAG‐3′	53	146
Calmodulin‐1	Sense 5′‐GGTCAGAACCCAACAGAAG‐3′	52	287
	Antisense 5′‐TGTCCGTCGCCATCAATAT‐3′		
Regucalcin	Sense 5′‐AGTTGGGAGGCTATGTTGC‐3′	52	378
	Antisense 5′‐TGCGGTTGGAAATCTGTC‐3′		
RAP2A	Sense 5′‐CCTGGTCGGGAACAAAGT‐3′	49	159
	Antisense 5′‐TTCATCTGCCGCACAATT‐3′		
*β*‐actin	Sense 5′‐ATATCGCTGCGCTGGTCGTC‐3′	58	517
	Antisense 5′‐AGGATGGCGTGAGGGAGAGC‐3′		

Abbreviations: MAP1B, microtubule‐associated protein 1B; MAP2, microtubule‐associated protein 2; MAPKK4, mitogen‐activated protein kinase kinase 4; RAB6A, Ras‐related protein RAB‐6A; RAP2A, Ras‐related protein RAP‐2A; RT‐PCR, reverse‐transcription polymerase chain reaction.

### 2.8 Molecular docking

To understand the effects of PNS and key genes, the three main components of PNS, Panaxytriol, Sanchinoside B1, and Notoginsenoside R4, were subjected to molecular docking with key core genes and screened differential genes. First, the two‐dimensional structure diagram of the drug monomer PNS was downloaded from the TCMIP database and analysis platform. Second, the three‐dimensional structure of the key target was obtained from the PDB database (https://www.rcsb.org/) and water molecules and small molecule ligands were deleted from the three‐dimensional structure of the key target by using PyMOL software. Third, AutoDock Vina 4.2.6 was used to conduct molecular docking between the macromolecular receptor protein and the small molecular ligand (drug monomer) to calculate the binding energy. It is generally believed that for protein–ligand complexes, the lower the binding energy, the higher the binding affinity. The results based on the binding energy of <5.0 kJ/mol indicated that the minimum binding energy of all selected bioactive components to the receptor was well below 5.0 kJ/mol. Finally, PyMOL software was used to visualize the model.

### 2.9 PPI recognition between Hub genes and experiment findings

To understand the relationship between Hub genes and differential genes, we used String database to analyze the PPI of eight Hub genes obtained through bioinformatics and four differential genes obtained from animal experiments.

### 2.10 Statistical methods

All data in this experiment were expressed as mean ± standard deviation, and SPSS21.0 statistical software was used for data processing. *T* test was performed on the expression level of each gene between the two groups, and *p* < 0.05 indicated that the difference had statistical significance.

## 3 RESULTS

### 3.1 Collection of PNS‐related genes and aging‐related genes

Through TCMIP and BATMAN‐TCM databases, we obtained 12 active compounds for PNS, which included Notoginsenoside R4, Ginsenoside Rb1, Notoginsenoside R3, Notoginsenoside R1, Notoginsenoside A, Acetophenone, Notoginsenoside R2, Sanchinoside B1, Cyclododecanone, Stigmasterol, Panaxytriol, and Cuparene (Supporting Information S1: Table 1). Then, 224 genes related to PNS were obtained through Swiss Target Prediction database (Supporting Information S1: Table 2), and 27,708 genes related to aging were obtained from the GeneCards database, and these genes were limited with the correlation degree ≥2, so as to acquire the last 5105 genes related to aging (Supporting Information S1: Table 3).

### 3.2 Intersecting genes between PNS and aging

**Figure 1:**
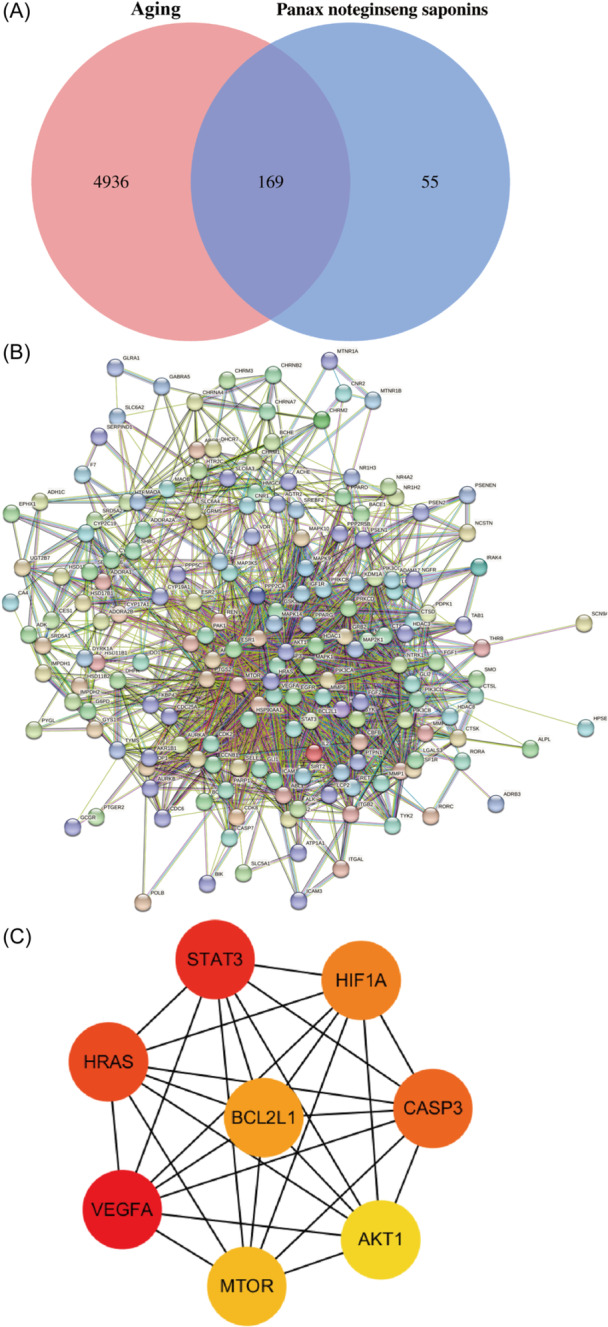
Screening of core targets. (A) Venn diagram of the intersecting targets of PNS and aging. The pink and blue circles, respectively, represent the screened aging and PNS targets, and the middle part represents the intersecting target of PNS and aging. (B) PPI network diagram of all intersecting genes. (C) PPI network diagram of the first eight Hub genes. The colors of Hub gene nodes were marked as red to yellow from high to low according to the Maximum Cross Correlation ranking. PNS, Panax notoginseng saponins; PPI, protein–protein interaction. [Color figure can be viewed at wileyonlinelibrary.com]

To harvest the cross genes, 224 genes related to PNS and 5105 genes related to aging were crossed and finally 169 intersection genes were obtained (Figure 1A, Supporting Information S1: Table 4). Moreover, these intersecting genes were subject to PPI analysis and the gene–gene interaction table was downloaded (Figure 1B, Supporting Information S1: Table 5), which was imported into Cytoscape3.7.2. to screen out the first eight Hub genes according to the Maximum Cross Correlation (MCC) ranking, designated as STAT3, VEGFA, HIF1A, CASP3, MTOR, BCL2L1, HRAS, and AKT1 (Figure 1C).

### 3.3 GO and KEGG analysis of intersecting genes

**Figure 2:**
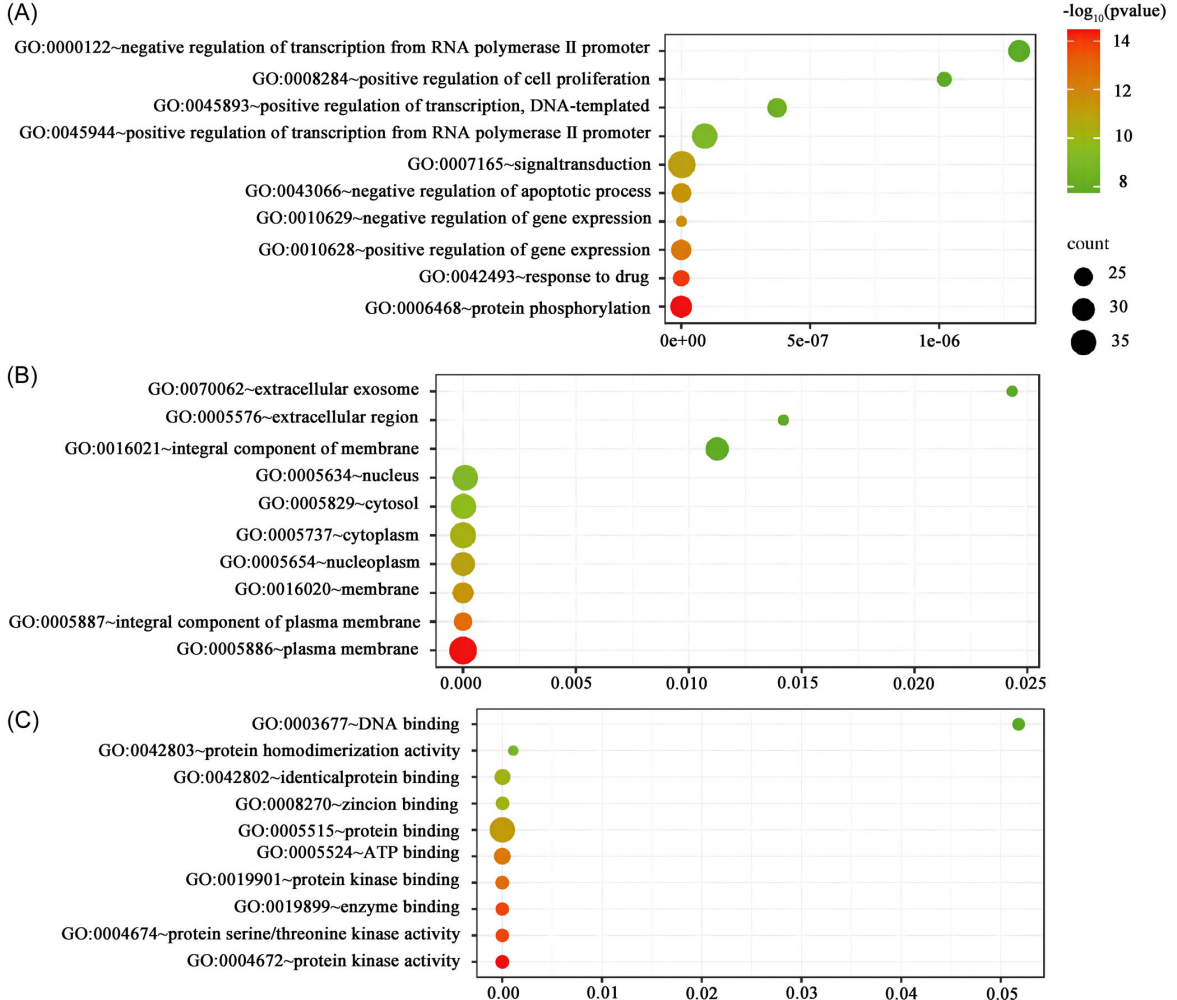
GO functional enrichment analysis. (A) Biological process of intersecting genes; (B) the cellular component of the intersecting gene; and (C) molecular functions of intersecting genes. The vertical coordinate was GO terms, the horizontal coordinate was GeneRatio, the horizontal coordinate represented pathway, and the vertical coordinate represented enrichment value. The size of bubbles indicated the number of enrichments in the pathway. The larger the bubble, the more enrichment would be conducted. The color would be redder, and the *p* value would be smaller, with higher reliability. GO, gene ontology. [Color figure can be viewed at wileyonlinelibrary.com]

**Figure 3:**
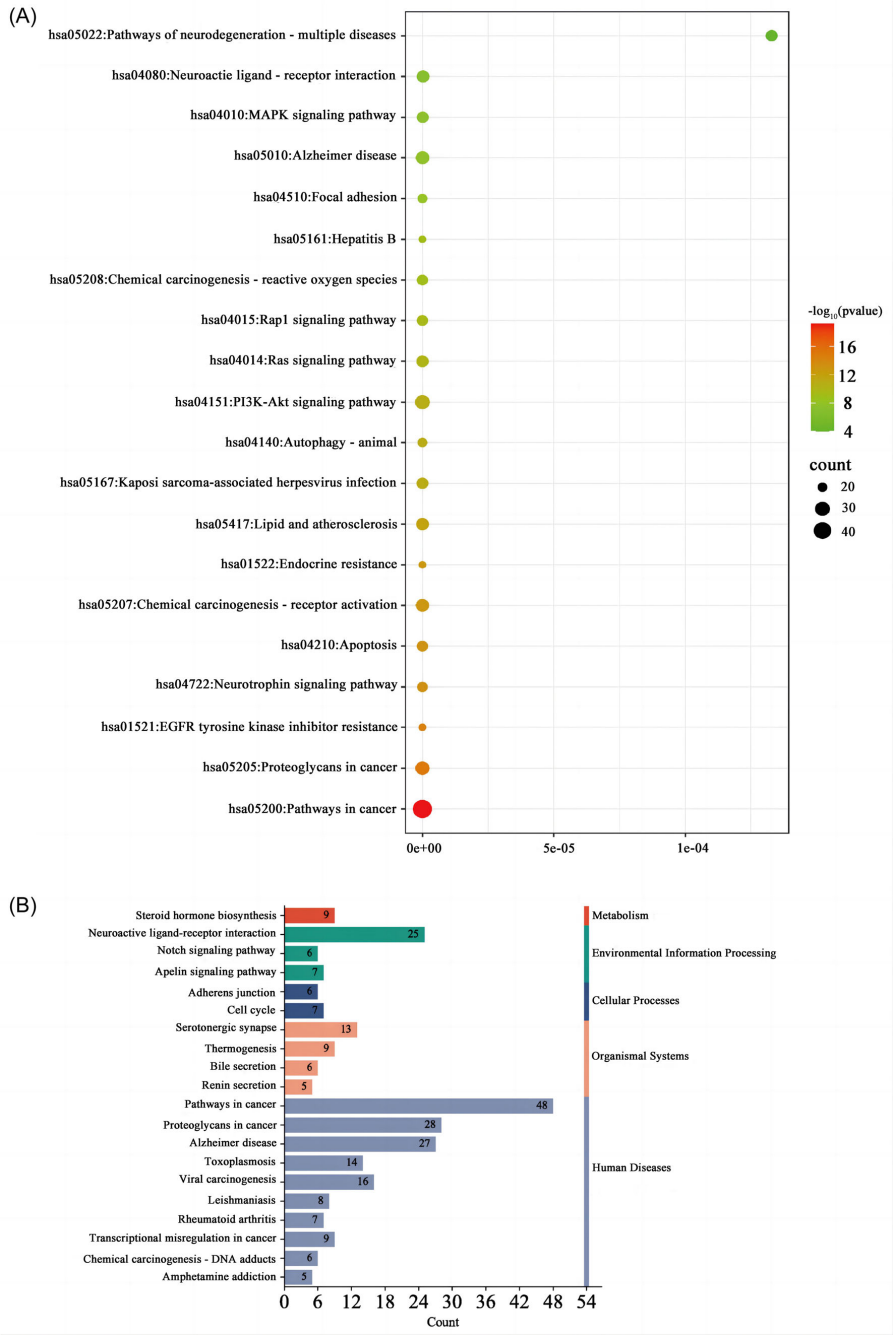
KEGG pathway enrichment analysis. (A) The horizontal axis represents GeneRatio (representing the ratio of the number of differential genes to the total number of differential genes under this pathway). The vertical axis indicates the pathway information enriched, and Count indicates the number of enriched genes. Different colors represent different adjusted *p* value, from blue to red, indicating that the adjusted *p* value increases from large to small and the enrichment degree is more and more significant. The size of the origin represents the number of genes enriched to this pathway. (B) The number of genes annotated to Routes A and B. In the figure, the vertical coordinate is the A‐level and B‐level classification of KEGG, the right vertical coordinate is the A‐level classification name, and the left vertical coordinate is the B‐level classification name. The abscissa is the number of genes on the corresponding B‐level classification. KEGG, Kyoto Encyclopedia of Genes and Genome. [Color figure can be viewed at wileyonlinelibrary.com]

GO analysis showed that BP mainly included the protein phosphorylation, response to drug, positive regulation of gene expression, negative regulation of gene expression, negative regulation of apoptotic process, signal transduction, positive regulation of transcription from RNA polymerase II promoter, positive regulation of transcription, DNA‐template, positive regulation of cell proliferation, and negative regulation of transcription from RNA polymerase II promoter (Figure 2A); CC mainly included plasma membrane, integral component of plasma membrane, membrane, nucleoplasm, cytoplasm, cytosol, nucleus, integral component of membrane, extracellular region, exosome (Figure 2B); MF mainly included the protein kinase activity, protein serum/thread kinase activity, enzyme binding, protein kinase binding, ATP binding, protein binding, Zinc binding, identical protein binding, Protein Homeodification Activity, and DNA binding (Figure 2C). According to the number of enriched passages, the top 20 were selected and a bubble chart was drawn. Then KEGG analysis showed that the signaling pathways of PNS and aging mainly involved pathways in cancer, proteoglycans in cancer, eGFR tyrosine kinase inhibitor resistance, neurotrophin signaling pathway, apoptosis, chemical carcinogenesis‐receptor activation, PI3K‐Akt signaling pathway, Ras signaling pathway, Rapl signaling pathway, and Alzheimer disease. The core pathways, including the MAPK signaling pathway, neuroactive ligand‐receptor interaction, and pathways of neurodegeneration multiple diseases, were potential therapeutic pathways for PNS to delay aging (Figure 3). The higher ranking suggested that it was more likely to be a key target pathway for antiaging of PNS (Supporting Information S1: Table 6).

### 3.4 Molecular docking of main components of PNS with core genes

**Figure 4:**
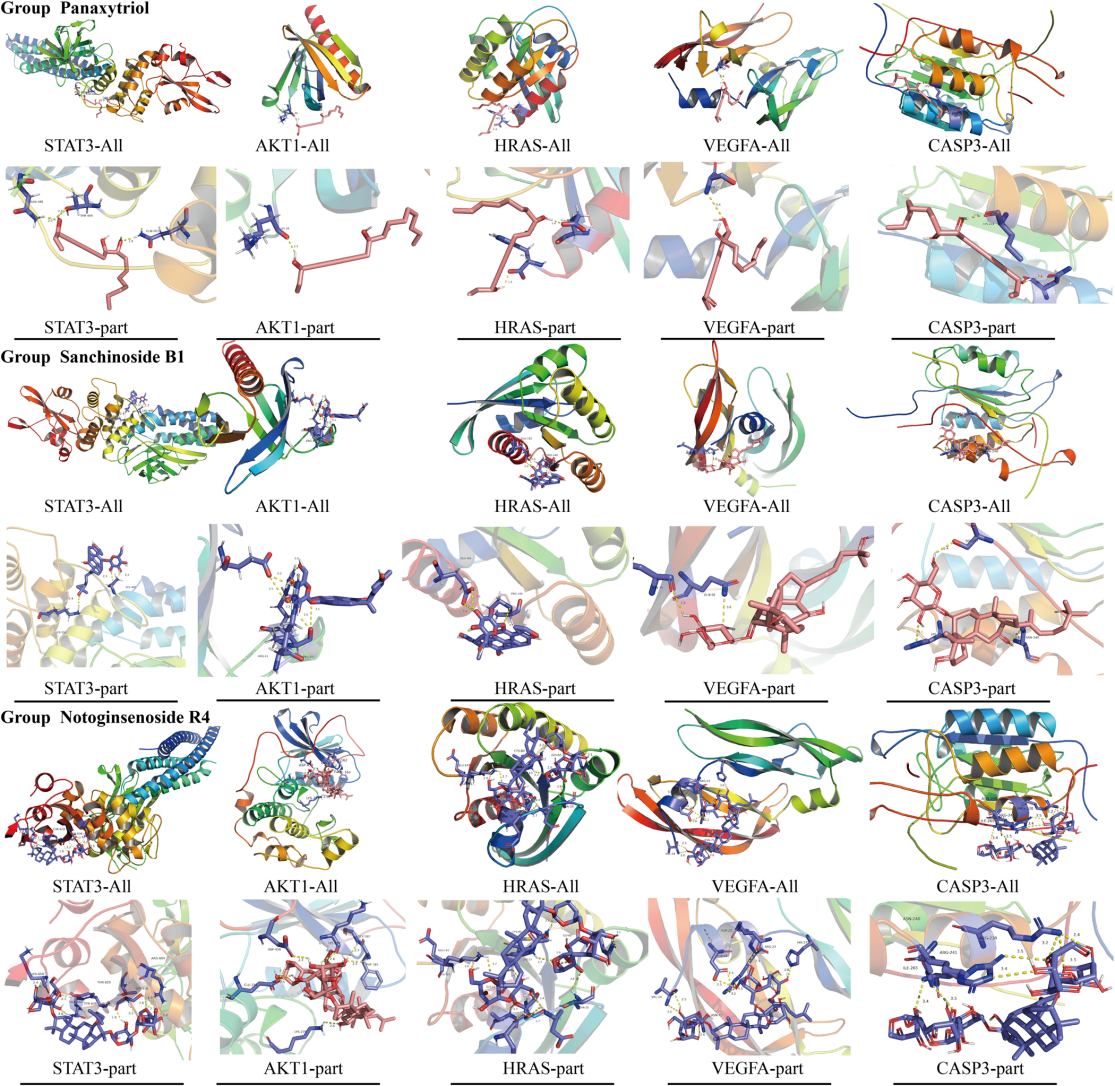
Molecular docking diagram of three main active substances and five core genes of PNS. Group Panaxytriol represented the molecular docking results of Panaxytriol with STAT3, AKT1, HRAS, VEGFA, and CASP3, respectively. Group Sanchinoside B1 indicated the molecular docking result diagram of Sanchinoside B1 with STAT3, AKT1, HRAS, VEGFA, and CASP3, respectively. Group Notoginsenoside R4 represents the molecular docking result diagram of Notoginsenoside R4 with STAT3, AKT1, HRAS, VEGFA, and CASP3, respectively. PNS, Panax notoginseng saponins. [Color figure can be viewed at wileyonlinelibrary.com]

Molecular docking was performed on the main active compounds of PNS, namely Panaxytriol, Sanchinoside B1, and Notoginsenoside R4, as well as the corresponding targets STAT3, AKT1, HRAS, VEGFA, and CASP3. The specific information for the binding energy of each active ingredient docked to each core target was shown in Table 2, and a visualization of docking results was shown in Figure 4. Molecular docking results showed that important bioactive components had good binding to core targets.

**Table 2:** Molecular docking binding energies of three major active substances of PNS and five core genes.

Active compound/core gene	STAT3	AKT1	HRAS	VEGFA	CASP3
Panaxytriol	−1.93	−2.16	−2.04	−4.9	−5.9
Sanchinoside B1	−2.91	−5.8	−4.41	−5.2	−6.4
Notoginsenoside R4	−8.20	−8.4	−9.00	−4.7	−5.1

Abbreviation: PNS, Panax notoginseng saponins.

### 3.5 Detection of PNS‐related differential genes

**Figure 5:**
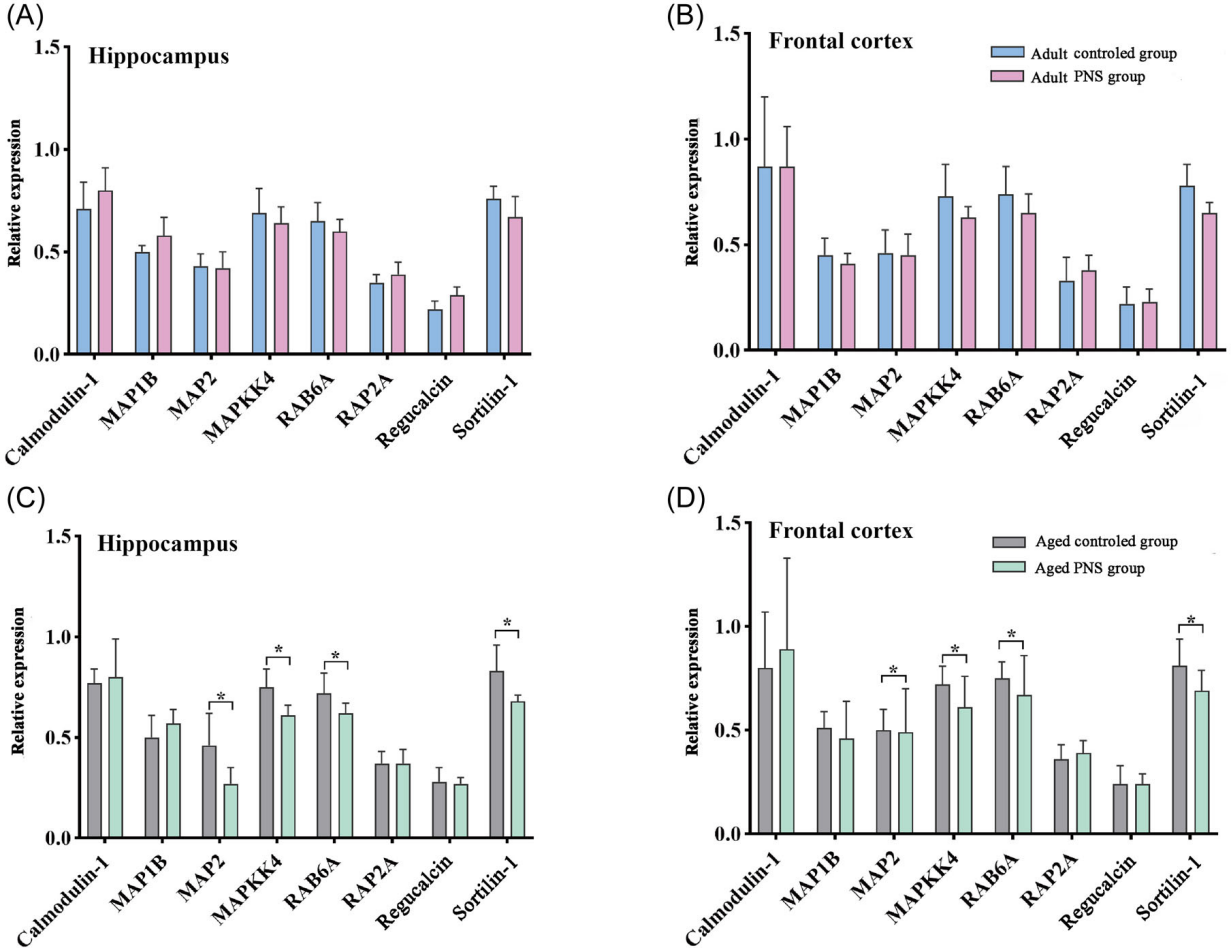
Distribution of aging‐associated genes in SAM‐P/8 mice. (A) Relative expression of genes in the hippocampus of the adult control group and the adult PNS group. (B) Gene expression in the frontal cortex of the adult control group and the adult PNS group. (C) The gene expression in hippocampus of aged control group and aged PNS group. (D) The gene expression in frontal cortex of aged control group and aged PNS group. PNS, Panax notoginseng saponins. *n* = 5; mean ± SD; *T* test; *p < 0.05, statistically significant. [Color figure can be viewed at wileyonlinelibrary.com]

The expression levels of eight aging‐related genes, including Calmodulin‐1, MAP1B, MAP2, MAPKK4, RAB6A, RAP2A, Regucalcin, and Sortilin‐1 in the hippocampus and frontal cortex, were detected in the adult control, adult PNS group, aged control group, and aged PNS group. RT‐PCR showed that they were not statistically significant when compared with the adult group. However, after the comparison between the aged PNS group and the aged control group, we found that the expressions of MAP2, RAB6A, Sortilin‐1, and MAPKK4 in the hippocampus were significantly decreased, with statistical significance (*p* < 0.05). Similar change was seen between the aged PNS group and the aged control group which revealed that the expressions of MAP2, RAB6A, Sortlin‐1, and MAPKK4 in the frontal lobe and all of them reported a statistical significance (*p* < 0.05) (Figure 5).

### 3.6 Molecular docking of main components of PNS and differential genes

**Figure 6:**
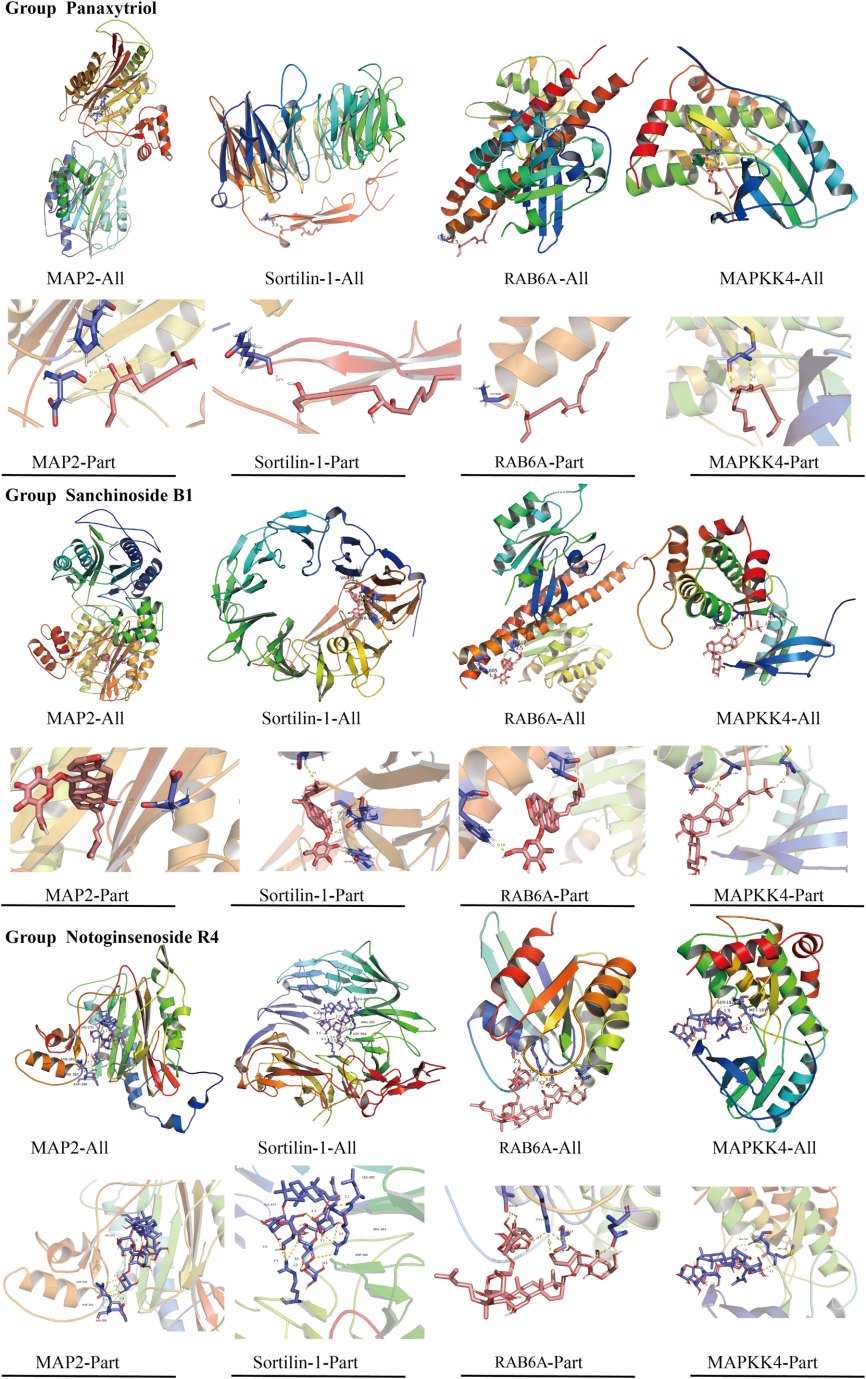
Molecular docking diagram of three main active substances of PNS and differential genes. Group Panaxytriol represented the molecular docking results of Panaxytriol with MAP2, Sortilin‐1, RAB6A, and MAPKK4, respectively. Group Sanchinoside B1 indicated the molecular docking result diagram of Sanchinoside B1 with MAP2, Sortilin‐1, RAB6A, and MAPKK4, respectively. Group Notoginsenoside R4 represents the molecular docking result diagram of Notoginsenoside R4 with MAP2, Sortilin‐1, RAB6A, and MAPKK4, respectively. PNS, Panax notoginseng saponins. [Color figure can be viewed at wileyonlinelibrary.com]

The main active compounds of PNS, panaxytriol, sanchinoside B1, and notoginsenoside R4, were molecular docked with the four differentially expressed genes MAP2, Sortilin‐1, RAB6A, and MAPKK4. Table 3 shows the specific information for the lowest binding energy of each active ingredient docked to each differential gene target. Figure 6 was the visualization of the docking results. Molecular docking results showed that important bioactive components had good binding abilities to differential genes.

**Table 3:** Molecular docking binding energies of three major active substances of PNS and differential genes.

Active compound/core gene	MAP2	Sortilin‐1	RAB6A	MAPKK4
Panaxytriol	−2.13	−1.62	−0.11	−6.0
Sanchinoside B1	−5.66	−3.47	−3.88	−5.3
Notoginsenoside R4	−4.2	−8.4	−5.7	−3.9

Abbreviation: PNS, Panax notoginseng saponins.

### 3.7 Relationship between eight Hub genes and four differential genes

**Figure 7:**
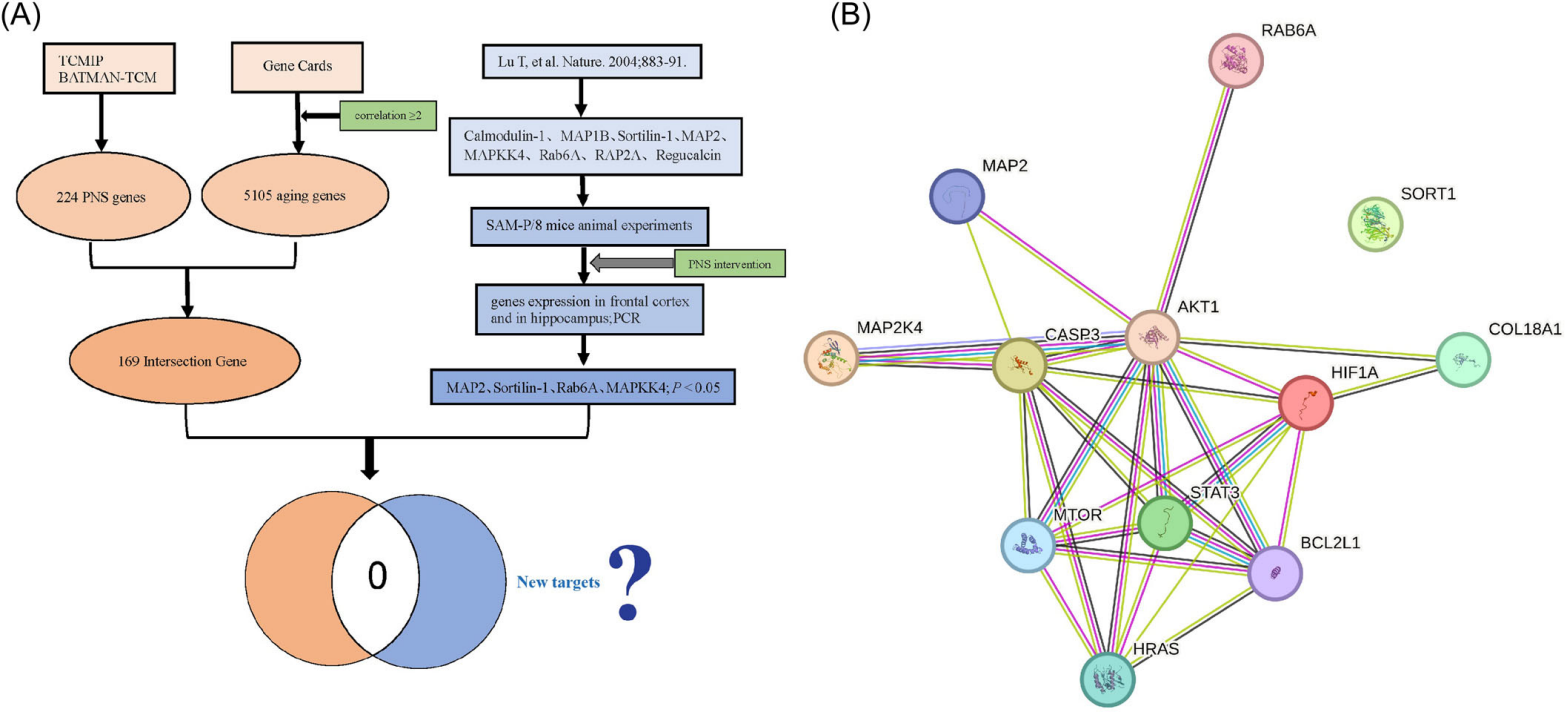
Relationship between Hub and differential genes. (A) The strategy to explore the relationship between intersection genes and differential genes. (B) Relationship of eight Hub genes with four differential genes using PPI. PCR, polymerase chain reaction; PNS, Panax notoginseng saponins; PPI, protein–protein interaction. [Color figure can be viewed at wileyonlinelibrary.com]

Using the above‐related gene platform, 169 genes after the intersection of PNS and aging genes were obtained. Eight aging‐related genes in the literature were selected for animal experiment verification. Under the intervention of PNS, RT‐PCR results showed that four genes, MAP2, Sortilin‐1, RAB6A, and MAPKK4, were low‐expressed in the frontal lobe and hippocampus in the aged PNS group compared with the aged control group. We intersected the obtained 169 intersecting genes with the four differential genes, but the results showed no intersection (Figure 7A). Next, we used String database to analyze the PPI so as to understand the association of the eight Hub genes on the four differential genes and found that among the differential genes, MAP2K4 was tightly associated with AKT1 and CASP3, MAP2 was associated with AKT1 and CASP3, RAB6A was associated with AKT1, and Sortlin‐1 (SORT1) did not have any association with the eight Hub genes (Figure 7B).

## 4 DISCUSSION

In this study, 169 intersecting genes related to PNS and aging were found by network pharmacology analysis, and five of the core genes were successfully molecular docked with the main components of PNS, which showed good binding activity. Second, animal experiments were performed to verify the aging‐related genes screened in the previous study, and four differential genes were obtained, which also showed good binding activity after molecular docking with the main component of PNS. At the same time, it was found that the 169 intersecting genes related to PNS and aging did not intersect with the four differential genes MAP2, Sortilin‐1, RAB6A, and MAPKK4.

AKT1 can be binded with PNS, which indicates that PNS may play an important role in the antiaging process by regulating AKT1. In addition, in the results of KEGG signaling pathway analysis, we also found that AKT1 was involved in the transmission of the first three signaling pathways, indicating that AKT1 was a key signal in the antiaging process of PNS. Literature shows that AKT1, a serine/threonine kinase Akt‐1, is the downstream target of phosphatidylinositol 3‐kinase (PI3K) and regulates cell proliferation,^17^ including cell proliferation, survival, metabolism, and angiogenesis of normal and malignant cells. Also, AKT1 is a key mediator of growth factor‐induced neuronal survival.^18^ Chalecka‐Franaszek and Chuang demonstrated that neuronal death by regulating the activation and phosphorylation of AKT1^19^ was reduced. In the research by Kulik et al., it was found that the endogenous IGF‐I receptor had antiapoptotic signal capability, while the overexpression of other tyrosine kinases enabled it to also have antiapoptotic capability, and the activation of PI3 kinase and Akt was sufficient to resist apoptotic signal.^20^ According to the current research, we have found that AKT1 plays the most important role in the neuroprotection and apoptosis signal transduction of Kang cells.^21^ In addition, AKT1 has been involved in EGFR tyrosine kinase inhibition resistance pathway, apoptosis, and neuroprotection. Together, AKT1 may be a key signaling molecule for PNS to play an antiaging role.

STAT3 and VEGFA were two of the eight key genes with the strongest correlation. According to our research results, we can conclude that STAT3 is involved in three of the first five signaling pathways, and VEGFA is involved in four of them. They are all involved in the cancer pathway, cancer proteoglycan pathway, and EGFR tyrosine kinase inhibition resistance pathway with significant significance. STAT3 is a signal transducer and transcriptional activator of factor 3, the gene‐encoding protein that is activated by phosphorylation in response to a variety of cytokines and growth factors. Various studies have unveiled the role of STAT3 in protecting the degeneration of human nucleus pulposus cells and reducing their apoptotic signaling pathways.^22^, ^23^ In addition, STAT3 has been found to have an effect of resisting various tumors and inhibiting cell proliferation and invasion, such as lung cancer,^24^ thyroid cancer,^25^ gastric cancer,^26^ and so on. Based on our research and previous reports, it can be demonstrated that STAT3 and VEGFA play key roles in the process of cell proliferation and differentiation, and the signaling pathways involved in these two molecules may play a key role in the antiaging process of PNS.

Animal experiments verified have differential the genes related to aging. Microtubule‐associated protein 2 (MAP2) is the main cytoskeletal regulator in neuronal dendrites, and it is abundant and specific enough to serve as a powerful marker of somatic dendritic cells, affecting microtubule dynamics and microtubule/actin interaction to control neurite growth and synaptic function.^27^ In the (human cytomegalovirus) HCMV‐UL122‐Tg mouse model, it was found to have more significant cognitive impairment than the 6‐month‐old mice, and the expression of MAP2 in the hippocampus was also reduced.^28^ To compare whether there are any significant changes in the hippocampal DTI index and MAP2 after ischemic treatment in adult and middle‐aged gerbils, the injury in middle‐aged gerbils is more serious than that in adult gerbils, which has confirmed that MAP2 can delay neuronal apoptosis and early dendritic injury.^29^ The study on human cerebral cortex samples showed that the expression of MAP2 gene was related to aging and AD progression,^30^ which was consistent with the research result in the literature.^16^ Sortilin is a key participant in the regulation of neuronal viability,^31^ as well as a variety of other biological functions, including cholesterol and glucose metabolism,^32^, ^33^ and it was found to decrease in the prefrontal cortex^34^ and hippocampus of aging rats,^35^ which were consistent with the results in the literature^36^ and our findings. Amazingly, the high expression of Sortilin‐1 can affect the reduction of neuronal apoptosis.^36^ Previous studies have shown that RAB6 has a potential neuroprotective effect, and the consumption of RAB6A in rat hippocampal neurons has been proved to reduce neurite growth.^37^ The results showed that RAB6A promoted the growth of neurites.^38^ It has been confirmed by mouse gene knockout experiments that the reduced expression of RAB6A can lead to microcephaly and neuronal dysplasia.^39^ However, there are reports that the level of RAB6A is elevated in the brain of AD patients,^40^ including the hippocampus, medial olfactory, and temporal lobe cortex.^41^ MAP2K4 is also known as JNKK, MEK4, MKK4, SEK1, SKK1, JNK1, SERK, MAPKK4, PRKMK4, and SAPKK1. As a member of mitogen‐activated protein kinase signaling system, MAPKK4 activates MAPK, phosphorylates nuclear transcription factors and other protein kinases and other substrates; regulates the transcription of related genes; participates in various physiological processes, such as cell growth, development, division, and functional synchronization between cells; and plays an important role in such pathological processes as apoptosis and malignant transformation.^42^ Tongqiao Huoxue Decoction (TQHXD) protects neurons from injury and prevents apoptosis by controlling ASK1/MKK4/JNK pathway.^43^ MKK4 knockdown may inhibit oxidative stress and subsequent apoptosis and play a protective role in hepatocyte injury.^44^ The deletion of MKK4 gene in adult mice leads to damage to hippocampal immature granulosa cells, thus affecting growth and development.^45^ MKK4 acts as a tumor suppressor,^46^ and upregulation of MAP2K4 activates the JNK signaling pathway to promote the growth of glioma cells.^47^ Overexpression of MAP2K4 inhibits effectively the neuroprotection effect of miRNA‐27a‐3p.^48^ Saikosaponin D inhibits proliferation and promotes apoptosis by activating the MKK4‐JNK signaling pathway in pancreatic cancer cells.^49^ Upregulating the expression of MAP2K4 in epilepsy can inhibit inflammatory response and hippocampal neuronal apoptosis;^50^ Paeoniflorin inhibits the apoptosis of hypothalamic neurons by inhibiting the MKK4‐JNK signaling pathway.^51^ Prenylated quinoline carboxylic acid derivatives prevent neuronal cell death by inhibiting MKK4;^52^ Inhibition of S‐nitrosation of MKK4 protects the hippocampal CA1 neurons in cerebral ischemia/reperfusion of rats;^53^, ^54^ MKK4 has also been identified as a key regulatory factor for liver regeneration.^55^ Whereas the neuroprotective effect of losartan on inhibition of brain MKK4‐related pathway in hippocampal CA1 region of rats was seen, and^56^ the reduction of skin carcinogenesis by inhibiting the expression of MAPKK4^57^ supported the vital role of MAPKK4 in cell proliferation. It is also reported in agriculture that the structure and seed size of rice could be regulated by MAPKK4.^58^ Certainly, MAPKK4 is an essential gene for the growth and reproduction of water chestnuts.^59^ It can also affect apoptosis by reducing the expression of MAPKK4.^60^ Increased expression of MAPKK4 was confirmed in human bladder cancer cell lines,^61^ and overexpression of MAPKK4 improved the drought tolerance of poplar.^62^

The aging genes excavated from bioinformatics did not intersect with the differential genes obtained from animal experiments. It was deduced that these four differential genes might be new action molecules of PNS for antiaging. In the next step, we can conduct experiments on the differential genes to further improve the research of PNS in aging. In addition, some key genes in our research have similar research significance in antiaging. This indicated that the key genes we analyzed might play a regulatory role in the key signaling pathways for the antiaging effect of PNS. In our study, we comprehensively analyzed the molecular action targets of antiaging effect of PNS and docked the five key genes with the strongest correlation among the main components with the differential genes verified by animal experiments and molecular docking. The results also showed that the key active components of PNS were well connected with the five core genes and four differential genes, which might suggest that the four differential genes should be new antiaging targets of PNS. Compared with previous studies, our study has the advantage of using big data for research, not only covering most of the gene molecules but also predicting and analyzing whether drugs can play a regulatory role. The analysis method not only saves resources but also obtains good effects, so as to provide good reference for current aging research and action mechanisms and drug development of AD and PD related to aging and has certain contributions to promote the development of refractory diseases. However, the specific mechanism of PNS and the antiaging effect of PNS need further research and analysis.

Together, in this study, we mined out the possible new targets of PNS antiaging effects through bioinformatics and network pharmacology, and the results showed that PNS could act as an antiaging agent through some of the aging genes, supported by experimental validation and relevant molecular docking, which has not been reported in previous studies.

## 5 CONCLUSION

We found that PNS can regulate the expression of different aging‐related genes, and act on different signaling pathways, to play an antiaging role, suggesting that the antiaging effect of PNS was achieved through the synergistic network and pathways. Notably, MAP2, Sortilin‐1, RAB6A, and MAPKK4 might be new antiaging targets, which will further expand the molecular targets of PNS in antiaging process.

## AUTHOR CONTRIBUTIONS

Yang‐Yang Zhao wrote the manuscript and performed some animal experiments. Li‐Xia Yang collected the data and performed some animal experiments. Shuang‐Yu Que and Lei‐Xing An analyzed the network pharmacology data and produced pictures. Abeer A. Teeti participated in the data analysis and article revision. Shun‐Wu Xiao participated in the experimental design and reviewed the article for revision.

## CONFLICT OF INTEREST STATEMENT

The authors declare no conflict of interest.

## ETHICS STATEMENT

Studies involving animals have been reviewed and approved by the Ethics Committee of Kunming Medical University, China (approval number: kmmu20230340).

## Supporting information

Supporting information.

## Data Availability

Data sets used and/or analyzed in this study may reasonably be requested from corresponding authors.
