# Supplementary material for: Systemic mechanism of Panax noteginseng saponins in antiaging based on network pharmacology combined with experimental validation
Source: Ibrain. 2024 Jun 1;10(4):519–35. doi: 10.1002/ibra.12165 (PMC11649391; doi:10.1002/ibra.12165)
Supplement: Supplementary file 1 — Supporting information. [file IBRA-10-519-s001.doc]

Supplementary Table 1. 12 Active components of PNS

| Chemical Component | Molecular formula | Number of corresponding genes |
| --- | --- | --- |
| Notoginsenoside R4 | C_59_H_100_O_27_ | 23 |
| Ginsenoside Rb1 | C_54_H_92_O_23_ | 23 |
| Notoginsenoside R3 | C_48_H_82_O_19_ | 23 |
| Notoginsenoside R1 | C_47_H_80_O_18_ | 23 |
| Notoginsenoside A | C_54_H_92_O_24_ | 10 |
| Acetophenone | C_8_H_8_O | 11 |
| Notoginsenoside R2 | C_41_H_70_O_13_ | 22 |
| Sanchinoside B1 | C_36_H_62_O_9_ | 37 |
| Cyclododecanone | C_12_H_22_O | 26 |
| Stigmasterol | C_29_H_48_O | 41 |
| Panaxytriol | C_17_H_26_O_3_ | 105 |
| Cuparene | C_15_H_22_ | 33 |

PNS, Panax notoginseng saponins

Supplementary Table 2. PNS gene table

| GSK3B | THRB | VEGFA | PIK3CD | HSD17B2 | PRKCD | HDAC10 | SREBF2 |
| --- | --- | --- | --- | --- | --- | --- | --- |
| METAP1 | AR | FGF1 | PIK3CB | HSD17B1 | ADK | CASP3 | ESR2 |
| CHRM4 | IGF1R | FGF2 | PIK3CG | MAP3K8 | ADORA2A | CASP7 | SERPINA6 |
| CHRNA4 | CA1 | HPSE | PIK3CA | EGFR | ADORA2B | PRKCB | PTPN1 |
| CHRM5 | CES1 | RORC | AGTR2 | SORD | SYK | MAP3K5 | RORA |
| CHRM2 | CES2 | PSEN2 | BCL2A1 | KDM5A | SMO | RET | BCHE |
| CHRM1 | CYP19A1 | HSP90AA1 | MAP2K1 | CCNB3 | GABRA5 | HTR1A | CYP2C19 |
| CHRM3 | CA4 | LGALS4 | APH1A | CCNB1 | MMP13 | MAPK8 | G6PD |
| CHRNA7 | NR1I3 | LGALS3 | PPM1B | CCNB2 | MMP9 | MAPK10 | CDC25A |
| ACHE | ADH1A | LGALS8 | PPP1CC | FKBP1A | MMP1 | CCNC | NR1H2 |
| CHRNB2 | PARP1 | BCL2L1 | PPP2CA | MTOR | TYK2 | CDK8 | VDR |
| ESR1 | MTNR1A | CDK1 | PPP2R5A | NTRK1 | ADAM17 | MAPK9 | PTGER1 |
| SLC6A2 | MTNR1B | HSD11B2 | NPC1L1 | AURKA | DYRK1A | PSEN1 | PTGER2 |
| HTR2C | TYMS | HSD11B1 | HIF1A | CSF1R | SELE | F2 | PPARD |
| SLC6A4 | SRD5A1 | ATP1A1 | MAPK14 | PDPK1 | AKT1 | TBXAS1 | GLRA1 |
| PTGS2 | ADH1C | PSENEN | UPP1 | KDR | BACE1 | AKR1B1 | SQLE |
| TACR2 | SRD5A2 | NCSTN | MAPK1 | ADORA1 | NR3C1 | PPARG | DHCR7 |
| ADRB3 | GLI2 | APH1A | GYS1 | CLK4 | NR1H3 | CTSD | PTPN6 |
| ADORA3 | GLI1 | PSEN1 | PAK1 | CLK1 | IRAK4 | REN | GCGR |
| SHBG | EPHX1 | APH1B | SIRT2 | CLK2 | ALK | CTSE | NOS2 |
| UGT2B7 | CTSK | PSEN2 | NR4A2 | DYRK1B | IMPDH1 | CYP2C9 | POLB |
| MAOB | SLC6A3 | PSENEN | CDK2 | AURKB | HDAC3 | ABCB11 | S1PR1 |
| MAOA | PAOX | NCSTN | ITGAL | CCNA2 | HDAC6 | GRB2 | IL2 |
| KDM1A | CTSL | APH1A | ICAM1 | CBFB | IMPDH2 | DHFR | F7 |
| S1PR5 | CTSB | PSEN1 | ITGB2 | CNR1 | HDAC5 | PTGFR | ALPL |
| SPHK2 | IDO1 | SLC5A2 | HRAS | CNR2 | HDAC8 | SLC29A1 | MAP3K14 |
| S1PR4 | STAT3 | SLC5A1 | PYGL | ABL1 | HDAC1 | CYP51A1 | MET |
| S1PR3 | PTAFR | TOP1 | SCN9A | GRM5 | HDAC11 | HMGCR | CYP17A1 |

PNS, Panax notoginseng saponins

Supplementary Table 3. Aging part gene table

| APOE | MT-TL1 | CFB | FBN1 | NBN | SHBG | GAPDH | DRD2 |
| --- | --- | --- | --- | --- | --- | --- | --- |
| PDGFRB | AKT1 | TGFB1 | PSEN1 | DMD | KDR | GHRL | FAS |
| TP53 | ERCC6 | HLA-DQB1 | FMR1 | GFAP | PPARGC1A | CHAT | HRAS |
| IGF1 | IL1B | ADIPOQ | BBS10 | CCND1 | KL | SCN1B | CYP2C19 |
| ABCA4 | BRAF | CFHR1 | CDH1 | IGFBP3 | IL1RN | IL18 | TH |
| CFH | ERBB2 | NFKB1 | C2 | KCNQ1 | FGFR3 | CYP1A1 | H19 |
| INS | IGF2 | ELN | F2 | CTSD | EGF | FTO | PCNA |
| IL6 | KRAS | SOD2 | CHEK2 | CYP19A1 | PSAP | ERCC4 | SDHB |
| BRCA1 | APP | NOS3 | JAK2 | CX3CR1 | APOA1 | PRNP | MT-ND1 |
| ARMS2 | ACE | AGER | FSCN2 | PTGS2 | DNMT3A | SERPINF1 | PALB2 |
| TNF | AR | MTOR | COL1A1 | RET | POLG | GLB1 | RETN |
| LMNA | TLR4 | PIK3CA | GSTM1 | CCL2 | GCK | NAMPT | SLC6A3 |
| BRCA2 | IGF1R | CXCL8 | ABCB1 | PON1 | FOXO3 | LPL | BMP2 |
| MAPT | CDKN2A | TERT | MPO | CYP2C9 | GSTT1 | GALC | SERPINA3 |
| FBLN5 | CFI | CTNNB1 | EPHA2 | VIM | AGT | NPPB | RAD51C |
| APOB | VDR | PRKN | GHR | FLT1 | PTPN11 | COL2A1 | BRIP1 |
| VEGFA | CST3 | COMT | BCL2 | REST | RAD51 | HTR2A | CACNA1A |
| HTRA1 | PTEN | GH1 | CYP3A4 | CAT | GABRG2 | OCA2 | MFSD8 |
| BDNF | HLA-DRB1 | MSH2 | F5 | MAPK1 | CLU | CNR1 | PLAU |
| EGFR | SNCA | GBA1 | SCN2A | GSTP1 | CTLA4 | CETP | HLA-B |
| WRN | MLH1 | SOD1 | HFE | SLC2A1 | TCF7L2 | ABCA1 | MIR146A |
| ALB | LEP | SIRT1 | HNF1A | HNF4A | ICAM1 | NFE2L2 | PPARG |
| MTHFR | MMP9 | SERPINE1 | ERCC1 | IL2 | AGTR1 | C9 | SCN1A |
| IL10 | CFHR3 | KCNJ11 | IFNG | APC | CYP1A2 | LDLR | INSR |
| HMCN1 | ERCC2 | LRRK2 | SLC6A4 | MECP2 | NF1 | ATXN2 | MSH6 |
| ESR1 | ATM | TP63 | SMAD4 | KIT | SMPD1 | CD36 | PPT1 |
| CRP | HIF1A | MMP2 | MDM2 | BGLAP | CRYAA | CYP2D6 | TPP1 |
| C3 | ACAN | ABCC8 | PARP1 | TTR | LIPC | PMS2 | FGF2 |

Supplementary Table 4. Intersection gene of PNS and Aging

| GSK3B | SRD5A1 | PSEN2 | EGFR | AKT1 | ABCB11 | F2 | CYP19A1 |
| --- | --- | --- | --- | --- | --- | --- | --- |
| CHRNA4 | ADH1C | SLC5A1 | CCNB1 | BACE1 | GRB2 | AKR1B1 | CA4 |
| CHRM2 | SRD5A2 | TOP1 | MTOR | NR3C1 | DHFR | PPARG | PARP1 |
| CHRM1 | GLI2 | F7 | NTRK1 | NR1H3 | HMGCR | CTSD | MTNR1A |
| CHRM3 | GLI1 | PIK3CD | AURKA | IRAK4 | CYP17A1 | REN | MTNR1B |
| CHRNA7 | EPHX1 | PIK3CB | CSF1R | ALK | SREBF2 | CYP2C9 | TYMS |
| ACHE | CTSK | PIK3CG | PDPK1 | IMPDH1 | ESR2 | MMP9 | PSEN1 |
| CHRNB2 | SLC6A3 | PIK3CA | KDR | HDAC3 | SERPINA6 | MMP1 | POLB |
| ESR1 | CTSL | AGTR2 | ADORA1 | HDAC6 | PTPN1 | TYK2 | CES1 |
| SLC6A2 | CTSB | BCL2A1 | AURKB | IMPDH2 | RORA | ADAM17 | CDK1 |
| HTR2C | IDO1 | MAP2K1 | CBFB | HDAC8 | BCHE | DYRK1A | ITGB2 |
| SLC6A4 | STAT3 | PPP2CA | CNR1 | HDAC1 | CYP2C19 | SELE | MMP13 |
| PTGS2 | IL2 | HIF1A | CNR2 | MET | G6PD | HRAS | MAPK9 |
| ADRB3 | VEGFA | MAPK14 | ABL1 | CASP3 | CDC25A | PYGL | NOS2 |
| SHBG | FGF1 | MAPK1 | GRM5 | CASP7 | NR1H2 | SCN9A | NCSTN |
| UGT2B7 | FGF2 | GYS1 | PRKCD | PRKCB | VDR | ALPL |  |
| MAOB | HPSE | PAK1 | ADK | MAP3K5 | PTGER2 | HSD17B2 |  |
| MAOA | RORC | SIRT2 | ADORA2A | RET | PPARD | HSD17B1 |  |
| KDM1A | HSP90AA1 | NR4A2 | ADORA2B | HTR1A | GLRA1 | HSD11B2 |  |
| THRB | LGALS4 | CDK2 | SYK | MAPK8 | DHCR7 | HSD11B1 |  |
| AR | LGALS3 | ITGAL | SMO | MAPK10 | PTPN6 | ATP1A1 |  |
| PSENEN | GCGR | CDK8 | GABRA5 | ICAM1 | BCL2L1 | IGF1R |  |

PNS, Panax notoginseng saponins

Supplementary Table 5. PPI network analysis

| **#node1** | **node2** | **coexpression** | **experimentally_determined_interaction** | **database_annotated** | **automated_textmining** | **combined_score** |
| --- | --- | --- | --- | --- | --- | --- |
| AKT1 | HSP90AA1 | 0.062 | 0.946 | 0.9 | 0.991 | 0.999 |
| AKT1 | MTOR | 0.081 | 0.904 | 0.9 | 0.991 | 0.999 |
| AR | HSP90AA1 | 0 | 0.884 | 0.9 | 0.989 | 0.999 |
| BCL2L1 | BIK | 0 | 0.879 | 0.9 | 0.941 | 0.999 |
| CCNB1 | CDK2 | 0.421 | 0.982 | 0.9 | 0.991 | 0.999 |
| CCNB1 | CDC25A | 0.729 | 0.715 | 0.9 | 0.896 | 0.999 |
| CCNB1 | CDK1 | 0.968 | 0.999 | 0.9 | 0.995 | 0.999 |
| CDC6 | CDK2 | 0.46 | 0.966 | 0.9 | 0.868 | 0.999 |
| DHFR | TYMS | 0.467 | 0.081 | 0.9 | 0.99 | 0.999 |
| EGFR | STAT3 | 0 | 0.931 | 0.9 | 0.989 | 0.999 |
| EGFR | PTPN1 | 0 | 0.893 | 0.9 | 0.988 | 0.999 |
| EGFR | GRB2 | 0 | 0.998 | 0.9 | 0.993 | 0.999 |
| EGFR | HSP90AA1 | 0 | 0.884 | 0.9 | 0.989 | 0.999 |
| ESR1 | IGF1R | 0 | 0.774 | 0.9 | 0.989 | 0.999 |
| ESR1 | HSP90AA1 | 0 | 0.887 | 0.6 | 0.989 | 0.999 |
| ESR1 | HDAC1 | 0 | 0.876 | 0.9 | 0.987 | 0.999 |
| F2 | SERPIND1 | 0.606 | 0.937 | 0.8 | 0.885 | 0.999 |
| FKBP4 | HSP90AA1 | 0.263 | 0.888 | 0.9 | 0.992 | 0.999 |
| FKBP4 | NR3C1 | 0 | 0.848 | 0.9 | 0.98 | 0.999 |
| GRB2 | LCP2 | 0.108 | 0.889 | 0.9 | 0.989 | 0.999 |
| GRB2 | MET | 0.052 | 0.807 | 0.9 | 0.989 | 0.999 |
| GRB2 | PTPN1 | 0.064 | 0.763 | 0.9 | 0.968 | 0.999 |
| HDAC1 | KDM1A | 0.123 | 0.969 | 0.5 | 0.992 | 0.999 |
| HDAC6 | HSP90AA1 | 0.053 | 0.885 | 0.9 | 0.991 | 0.999 |
| HIF1A | STAT3 | 0.062 | 0.839 | 0.9 | 0.968 | 0.999 |
| HIF1A | HSP90AA1 | 0 | 0.918 | 0.9 | 0.991 | 0.999 |
| HRAS | PIK3CA | 0.062 | 0.871 | 0.9 | 0.976 | 0.999 |
| HSP90AA1 | PPP5C | 0.106 | 0.965 | 0.9 | 0.933 | 0.999 |
| HSP90AA1 | NR3C1 | 0 | 0.923 | 0.9 | 0.989 | 0.999 |
| HSP90AA1 | STAT3 | 0 | 0.27 | 0.9 | 0.988 | 0.999 |
| ICAM1 | ITGAL | 0.091 | 0.972 | 0.9 | 0.989 | 0.999 |
| ICAM1 | ITGB2 | 0.113 | 0.835 | 0.9 | 0.989 | 0.999 |
| ICAM3 | ITGAL | 0.172 | 0.932 | 0.9 | 0.986 | 0.999 |
| ITGAL | ITGB2 | 0.418 | 0.87 | 0.9 | 0.989 | 0.999 |
| KDR | VEGFA | 0.062 | 0.984 | 0.9 | 0.992 | 0.999 |
| MAPK14 | TAB1 | 0 | 0.966 | 0.9 | 0.987 | 0.999 |
| NCSTN | PSEN1 | 0.083 | 0.989 | 0.9 | 0.994 | 0.999 |
| NCSTN | PSEN2 | 0.063 | 0.839 | 0.8 | 0.994 | 0.999 |
| NCSTN | PSENEN | 0.062 | 0.987 | 0.9 | 0.995 | 0.999 |
| NGFR | NTRK1 | 0.062 | 0.865 | 0.9 | 0.99 | 0.999 |
| PPP2CA | PPP2R5B | 0.062 | 0.996 | 0.8 | 0.83 | 0.999 |
| PSEN1 | PSENEN | 0.062 | 0.982 | 0.9 | 0.993 | 0.999 |
| PSEN2 | PSENEN | 0.062 | 0.908 | 0.8 | 0.976 | 0.999 |

PPI, protein-protein interaction

Supplementary Table 6. Anti-aging KEGG signaling pathway of PNS

| Term | ID | Count | P Value | Genes |
| --- | --- | --- | --- | --- |
| Pathways in cancer | hsa05200 | 48 | 7.32E-20 | RET/ ALK/ CSF1R/ GSK3B/ HDAC1/ PTGER2/ PIK3CD/ PIK3CB/ FGF1/ PTGS2/ GLI1/ FGF2/ HIF1A/ EGFR/ IGF1R/ GLI2/ MAPK9/ CASP7/ MAPK8/ CASP3/ ABL1/ AKT1/ MAPK1/ HRAS/ NTRK1/ MAP2K1/ HSP90AA1/ NOS2/ PRKCB/ MMP1/ STAT3/ F2/ MMP9/ ESR1/ IL2/ MTOR/ ESR2/ VEGFA/ MAPK10/ AR/ SMO/ PIK3CA/ CDK2/ GRB2/ PPARG/ MET/ BCL2L1/ PPARD |
| Proteoglycans in cancer | hsa05205 | 28 | 1.11E-15 | PIK3CD/ PIK3CB/ FGF2/ HIF1A/ EGFR/ IGF1R/ PAK1/ CTSL/ CASP3/ KDR/ AKT1/ MAPK1/ HRAS/ MAP2K1/ PRKCB/ PDPK1/ STAT3/ MAPK14/ MMP9/ ESR1/ MTOR/ VEGFA/ SMO/ PIK3CA/ GRB2/ PTPN6/ HPSE/ MET |
| Alzheimer disease | Hsa05011 | 27 | 1.55E-08 | CHRM3/ GSK3B/ CHRM1/ CHRNA7/ PSEN2/ PIK3CD/ PSEN1/ PIK3CB/ PTGS2/ MAPK9/ CASP7/ NCSTN/ MAPK8/ GRM5/ CASP3/ AKT1/ MAPK1/ HRAS/ MAP3K5/ PSENEN/ MAP2K1/ NOS2/ MTOR/ BACE1/ MAPK10/ ADAM17/ PIK3CA |
| Neuroactive ligand-receptor interaction | hsa04080 | 25 | 8.86E-08 | CHRM2/ CHRM3/ THRB/ CHRM1/ CHRNA4/ PTGER2/ CHRNA7/ HTR2C/ NR3C1/ GLRA1/ GRM5/ CNR2/ CNR1/ ADORA1/ CHRNB2/ GABRA5/ GCGR/ HTR1A/ F2/ MTNR1A/ ADORA2A/ ADRB3/ MTNR1B/ ADORA2B/ AGTR2 |
| Pathways of neurodegeneration - multiple diseases | hsa05022 | 23 | 1.05E-04 | CHRM3/ GSK3B/ MAP2K1/ CHRM1/ NOS2/ PRKCB/ CHRNA7/ PSEN2/ PSEN1/ PTGS2/ MAPK14/ SLC6A3/ MTOR/ MAPK10/ MAPK9/ GRM5/ CASP7/ MAPK8/ CASP3/ MAPK1/ HRAS/ BCL2L1/ MAP3K5 |

KEGG, Kyoto Encyclopedia of Genes and Genome; PNS, Panax notoginseng saponins
